# Impact of left atrial appendage morphology on thrombus formation in TAVI patients with atrial fibrillation

**DOI:** 10.1186/s40001-023-01057-y

**Published:** 2023-02-20

**Authors:** N. Abanador-Kamper, J. Bepperling, M. Seyfarth, P. Haage, L. Kamper

**Affiliations:** 1 Cardiology & Heart Center, HELIOS University Hospital Wuppertal, University Witten/Herdecke, Witten, Germany; 2 Gynaecology, Marien Hospital Witten, University Witten/Herdecke, Witten, Germany; 3 Diagnostic & Interventional Radiology, HELIOS University Hospital Wuppertal, University Witten/Herdecke, Heusnerstr. 30, 42883 Wuppertal, Germany

**Keywords:** Left atrial appendage, Atrial fibrillation, Risk assessment, Thrombus, Stroke

## Abstract

**Purpose:**

We aimed to correlate left atrial appendage (LAA) morphology with thrombus formation in patients with severe aortic valve stenosis and atrial fibrillation.

**Methods:**

We analyzed LAA morphology and the prevalence of a thrombus in 231 patients with atrial fibrillation and severe aortic valve stenosis that were referred for pre-interventional CT scan before trans-catheter aortic valve implantation (TAVI) between 2016 and 2018. In addition, we documented neuro-embolic events depending on the presence of LAA thrombus within a follow-up of 18 months.

**Results:**

The overall distribution of different LAA morphologies was chicken-wing 25.5%, windsock 51.5%, cactus 15.6% and cauliflower 7.4%. Compared to chicken-wing morphology, patients with non-chicken-wing morphology showed a significantly higher thrombus rate (OR: 2.48, 95%; CI 1.05 to 5.86, *p* = 0.043). Within the 50 patients with a LAA thrombus, we observed chicken-wing (14.0%), windsock (62.0%), cactus (16.0%) and cauliflower (8.0%) configuration. In patients with LAA thrombus those with chicken-wing configuration have a higher risk (42.9%) to develop neuro-embolic events compared to non-chicken-wing configuration (20.9%).

**Conclusion:**

We found a lower LAA thrombus rate in patients with chicken-wing morphology compared to patients with non-chicken-wing configuration. However, in the presence of thrombus, those patients with chicken-wing morphology showed a doubled risk for neuro-embolic events compared to patients with non-chicken-wing morphology.

These results must be confirmed in larger trials but underline the importance of LAA evaluation in thoracic CT scans and could have an impact on the anticoagulation management.

## Introduction

Cardio-embolic strokes are a primary cause of mortality and a leading cause of long-term disability worldwide [1]. With over 90% the left atrial appendage (LAA) is the major thrombus location [2, 3], especially in patients with atrial fibrillation (AF). Johnson et al. referred to the LAA as most lethal human detachment [4]. It derives from the primordial left atrium and functions as a reservoir during conditions of fluid overload. The LAA shape is highly variable and has been classified in different schemes [5]. However, the shape and structure’s imaging is of profound importance to detect a thrombus of the LAA [5]. Cardiac CT has a high diagnostic accuracy for the detection of cardiac thrombi [6]. The aim of the study was to determine an association between the LAA morphology, the occurrence of LAA thrombus and potentially resulting neuro-embolic events.

## Material and methods

### Study population

**Fig. 1:**
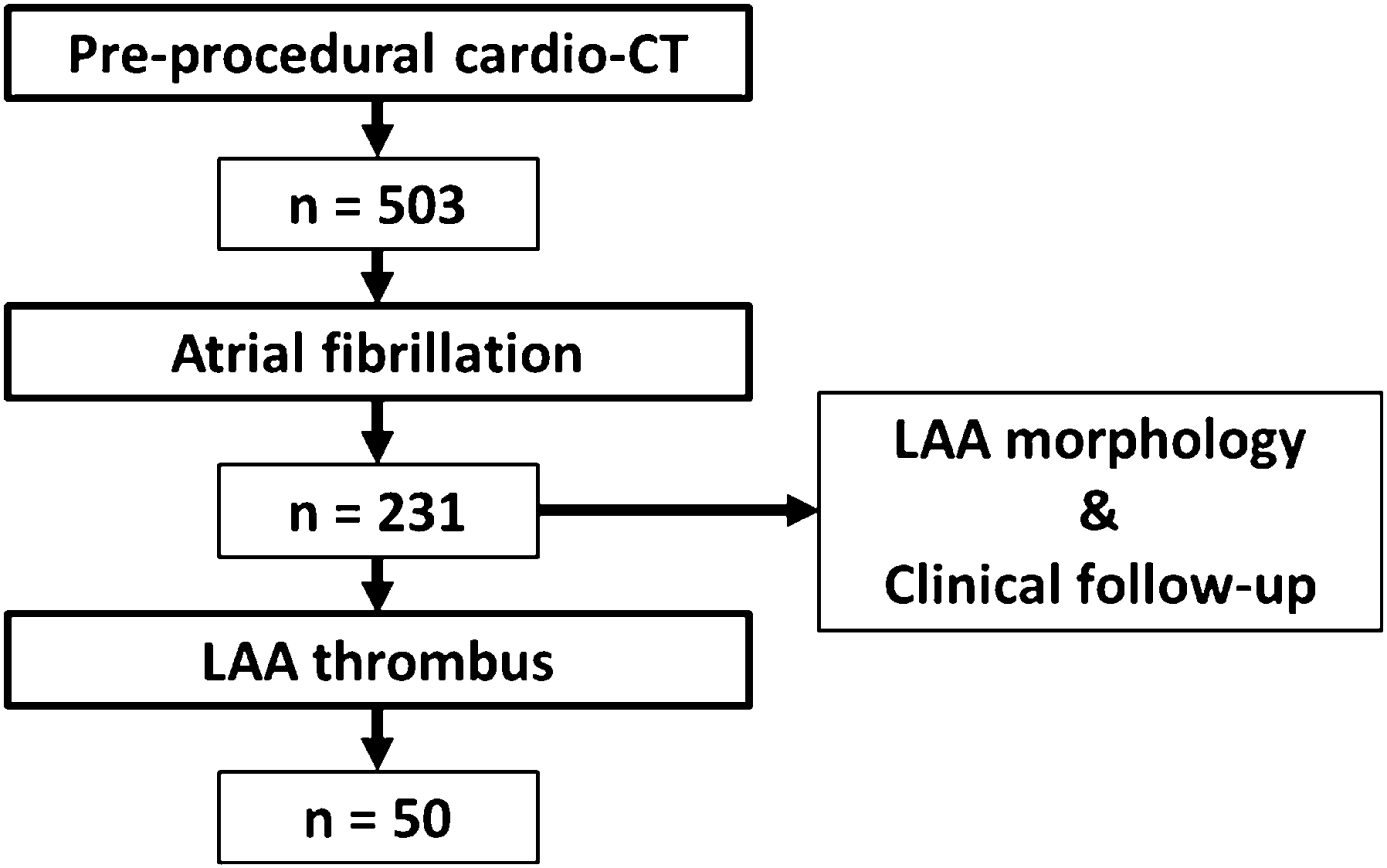
The flow chart displays the analyzed patients and those with detected LAA thrombus

This single-centre cross-sectional study retrospectively included patients with symptomatic aortic valve stenosis (AS) that underwent a pre-procedural CT scan before trans-catheter aortic valve implantation (TAVI). We screened 503 patients between January 2016 and January 2018 at a local university hospital. Entry criteria included existing pre-procedural CT scan between 1st of January 2016 and 1st of January 2018 and diagnosed atrial fibrillation. The resulting collective consisted of 231 patients (flow chart in Fig. 1). The patients’ medical histories were retrospectively collected using their chart reviews in the clinical information system (SAP, Walldorf, Germany). Age, gender, BMI, history of hypertension, diabetes, heart failure, cardiomegaly, aortic valve replacement (AVR), and the presence of a cardiac pacemaker were recorded. CHA2DS2-VASc scores were calculated with one point assigned for a history of congestive heart failure, hypertension, and 74 years ≥ age ≥ 65 years, female, diabetes mellitus and vascular disease and two points assigned for age ≥ 75 years, a history of stroke or transient ischemic attack (TIA), with a maximum score of nine. Demographic and clinical data are given in Table 1. The study was approved by our institutional review board. Patients gave written informed consent for the clinical examinations.

**Table 1:** Baseline characteristics of the study population

	overall population (*n* = 231)	patients with a thrombus (*n* = 50)	patients without a thrombus (*n* = 181)	*p*-value
Age	83 ± 5.6	85 ± 4.3	83 ± 5.8	*0.086*
BMI	26 ± 5.2	25 ± 4.1	26 ± 5.4	*p* < *0.05*
Male	120 (51.9%)	30 (60.0%)	90 (49.7%)	0.206
Dyslipidaemia	121 (52.5%)	17 (34.0%)	104 (57.5%)	*p* < *0.05*
Hypertension	215 (93.1%)	46 (92.0%)	169 (93.4%)	0.755
Diabetes	67 (29.0%)	13 (26.0%)	54 (29.8%)	0.725
Heart failure	121 (52.4%)	27(54.0%)	94 (51.9%)	0.873
CHA2DS2-VASc	5 ± 1.1	5 ± 1.0	5 ± 1.1	0.522
LA diameter	53 ± 8.0	55 ± 6.0	52 ± 8.4	*0.162*
Cardiomegaly	57 (24.7%)	18 (36.0%)	39(21.5%)	*p* < *0.05*
Anticoagulants	158 (68.4%)	36 (72.0%)	122 (67.4%)	*0.608*

### CT protocol

All patients underwent a retrospectively ECG-gated spiral MDCT of the aortic valve region (Somatom Definition Flash, Siemens Medical, Erlangen, Germany). The CT scans were performed with a tube voltage of 100 kV and a tube current of 300 mAs. We used Ultravist 300 (Bayer, Berlin, Germany) as intravenous contrast agent. The injection protocol included a test bolus of 15 ml, followed by an injection of 95 ml with an injection rate of 6.0 ml/s. Images were reconstructed by an iterative reconstruction kernel (I39f) with a slice thickness of 0.75 mm with automated detection of best-systolic and best-diastolic time-points of the R–R interval. Additional reconstructions were performed at every ten percent of the R–R interval.

### Image analysis

**Fig. 2:**
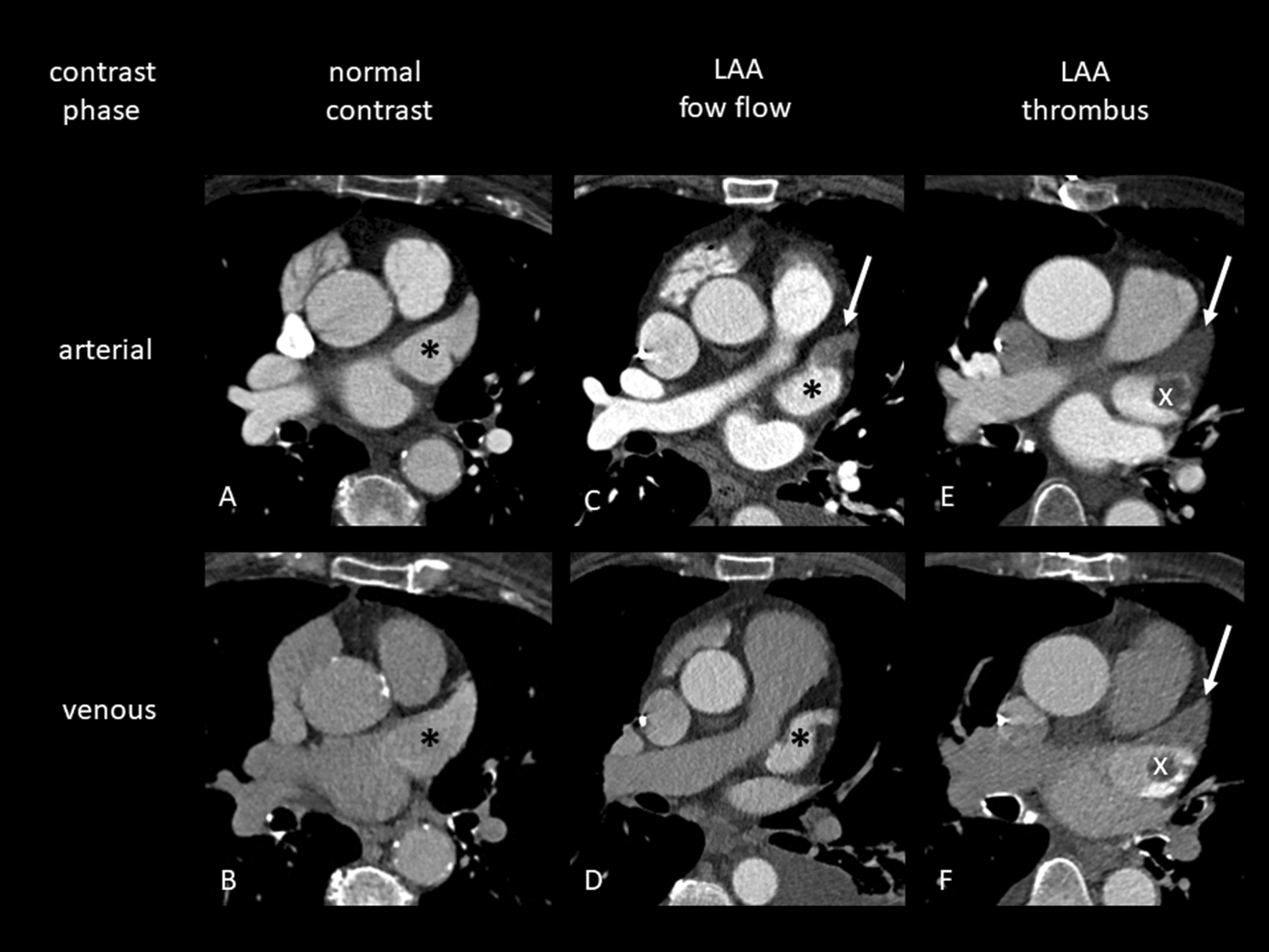
Detection of normal contrast, LAA low flow and LAA thrombus in MDCTNormal contrast of the LAA (*) in the arterial and venous contrast phase. LAA filling defect (arrow) in the arterial phase with regular venous contrast. Thrombus in the LAA apex (arrow) together with a rotund thrombus in the proximal LAA (x) identified by persistent filling defect in arterial and venous contrast phase.

**Fig. 3:**
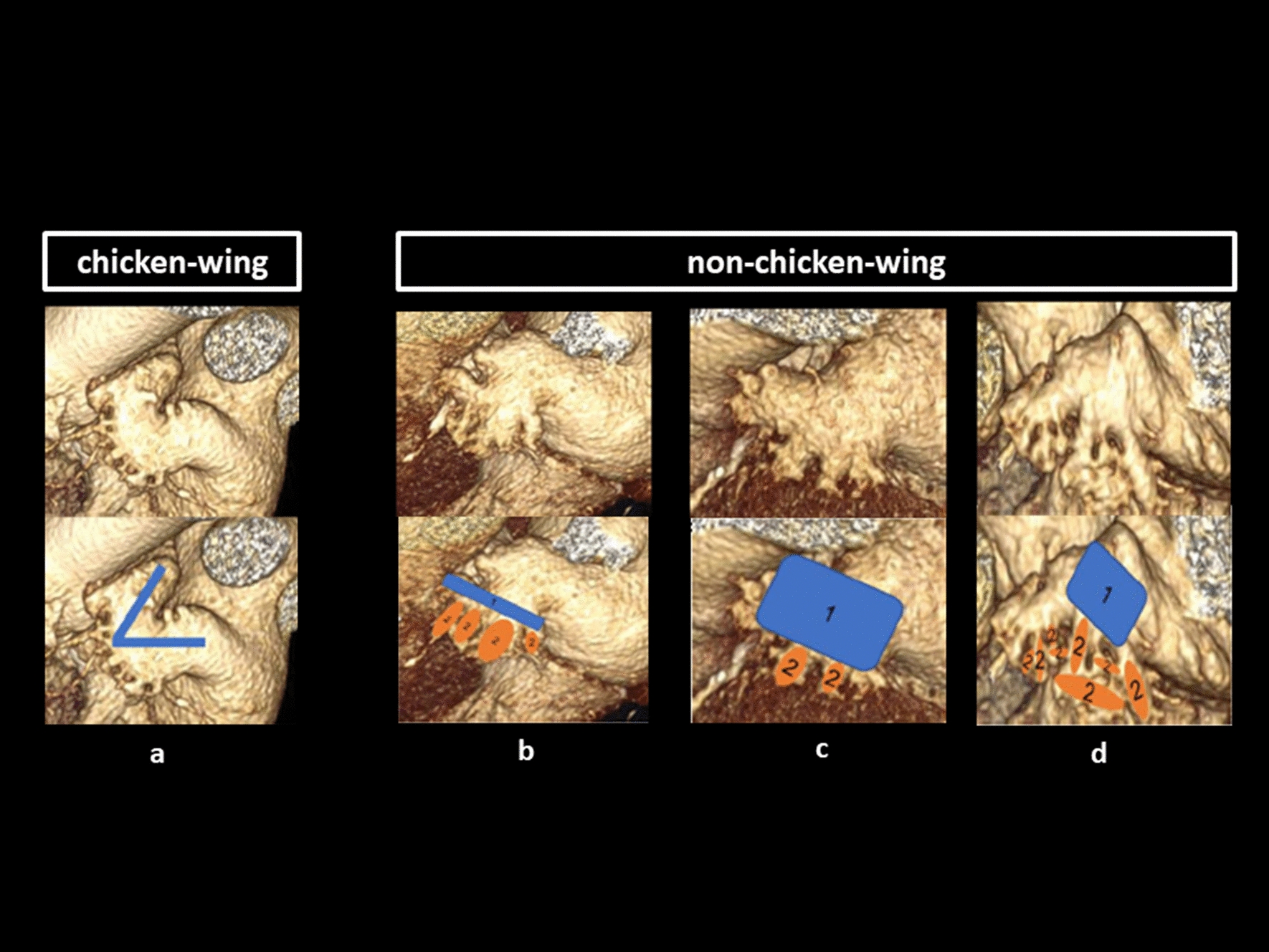
LAA classification with chicken-wing (**A**) and non-chicken-wing configurations (**B**–**C**). Chicken-wing configuration (**a**) is defined by a noticeable bend in the dominant lobe’s proximal or middle part. Windsock configuration (**b**) has a dominant lobe of sufficient length and limited width (1) and variable location and number of secondary lobes (2). Cactus configuration (**c**) shows a dominant central lobe (1) and varying secondary lobes (2). Compared to windsock, the dominant lobe is sufficient of width and comparatively limited in length. Cauliflower configuration (**d**) shows a limited length and more complex internal characteristics (2) sometimes even without a dominant lobe (1)

CT images were stored in our picture archiving system (Centricity, GE-Healthcare, Milwaukee, WI, USA). Image analysis of arterial and venous phase images was performed in consensus by two experienced examiners. LAA thrombus was defined as a persistent and significant filling defect in both contrast phases (Fig. 2). The left atrial diameter was assessed in the axial plane vertically to the aortic valve. We categorized the LAA anatomy into four different morphologies (Fig. 3): chicken-wing, windsock, cauliflower, and cactus, based on Wang’s classification [5].

### Follow-up

We documented neuro-embolic events depending on the LAA thrombus’s presence within a follow-up of 18 months after TAVI. Post-procedural neuro-embolic events were defined as a hospital re-admission due to an acute, ischemic, neurological event within 18 months after the pre-procedural CT scan.

### Statistical analysis

All statistical analyses were performed by SPSS for Windows version 24 (SPSS Inc., Chicago, IL). All continuous data are presented as the mean ± SD and were compared using Student’s t-test. Categorical variables are described as count and percent and were compared using Fisher’s exact test. The odds ratio (OR) and 95% confidence interval (CI) of OR for LAA thrombus were computed. All tests were 2-sided, and a *p*-value < 0.05 was considered statistically significant.

## Results

**Fig. 4:**
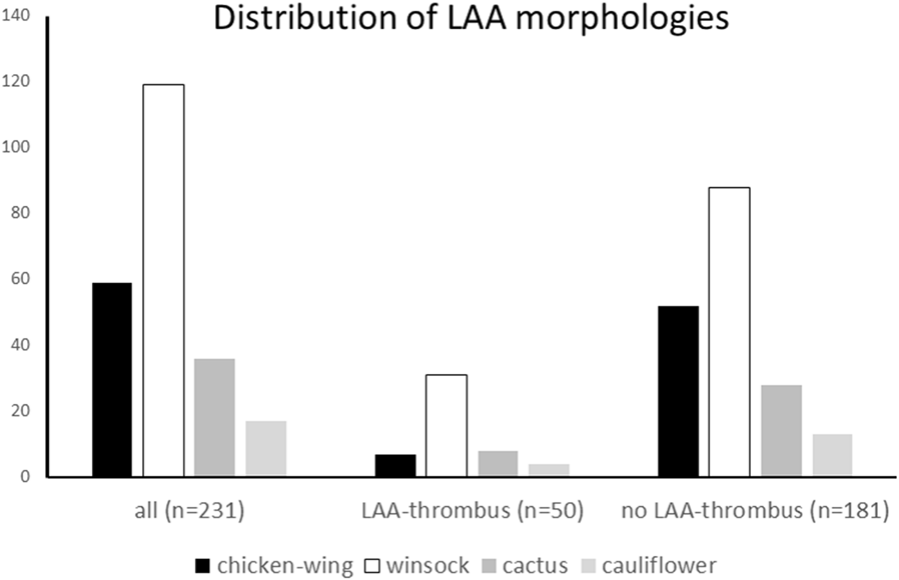
Distribution of LAA configurations for all patients and those with and without LAA thrombus

**Fig. 5:**
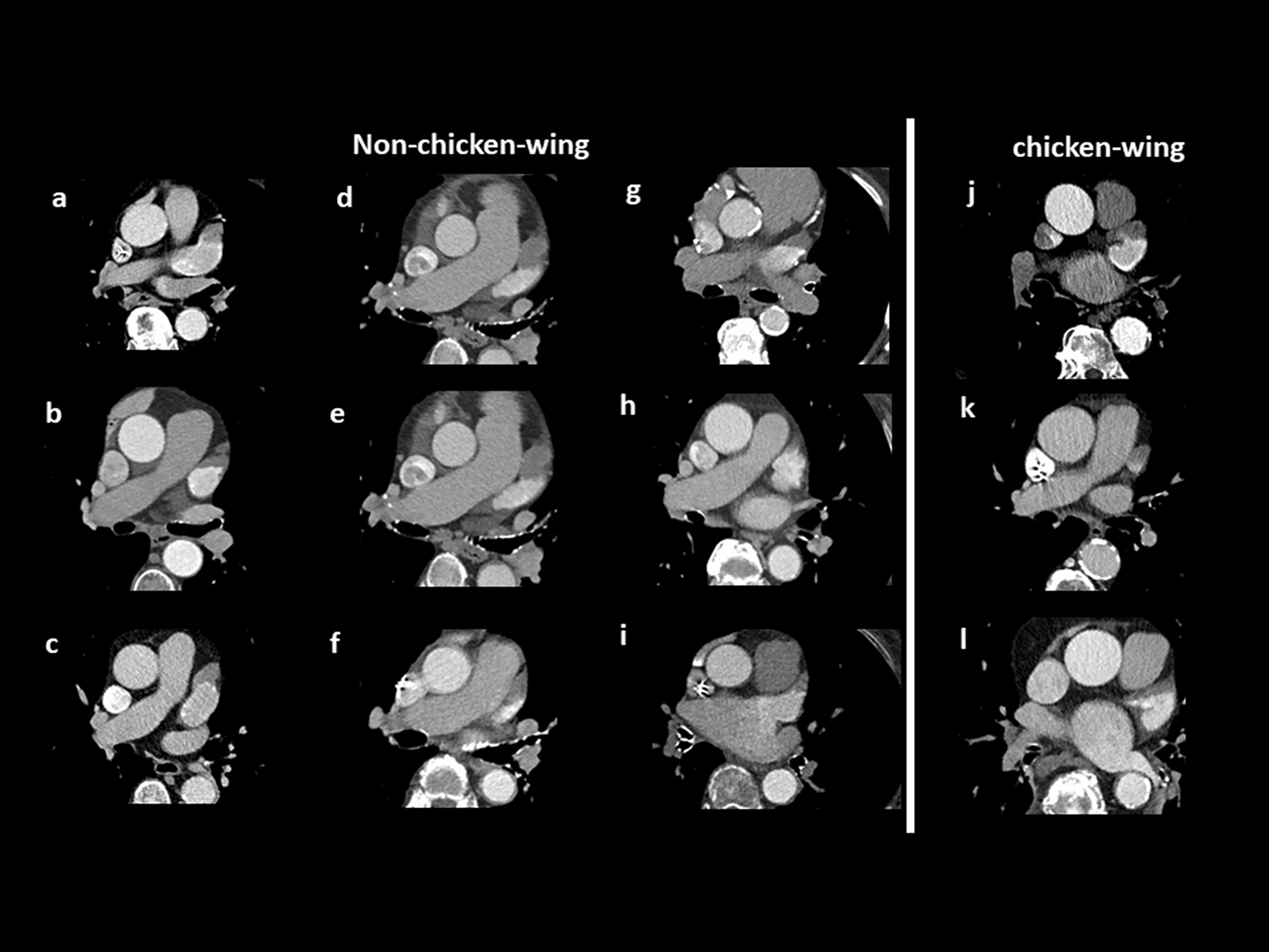
Exemplary images of patients with LAA thrombus and documented neuro-embolic event within 18 months after the TAVI-procedure, categorized in non-chicken-wing (**a**–**i**) and chicken-wing LAA configuration (**j**–**l**)

We examined CT scans of 231 patients (age 83 ± 5.6, 51.9% were male, CHA2DS2-VASc Score 5 ± 1.1). Table 1 presents the baseline and clinical characteristics. The distribution of chicken-wing, windsock, cactus and cauliflower configuration is given in Fig. 4. In the overall collective windsock configuration was found in 119 of the patients (51.5%) and chicken-wing in 59 (25.5%). LAA thrombus was diagnosed in 50 (21.6%) patients and was excluded in 181 (79.4%) patients. The two groups showed significantly differences for BMI, dyslipidaemia, cardiomegaly, and chicken-wing configuration (Table 2). No significant differences were found in age, gender, hypertension, diabetes mellitus, heart failure, CHA2DS2-VASc Score, pacemaker, and left atrium diameter. Within the 50 patients with LAA thrombus, the distribution was chicken-wing 14.0%, windsock 62.0%, cactus 16.0%, and cauliflower 8.0% (Fig. 4). Chicken-wing configuration was significant (*p* < 0.05) more prominent in patients with thrombus exclusion (Table 2). Compared to chicken-wing morphology, patients with non-chicken wing morphology were significantly more likely to have a thrombus (OR: 2.48, 95% CI 1.05 to 5.86, *p* = 0.043). Figure 5  provides exemplary images of patients with LAA thrombus.

**Table 2:** Distribution of the LAA Configuration in all patients, for patients with detected LAA thrombus and for those with excluded LAA thrombus

	overall population (*n* = 231)	LAA thrombus (*n* = 50) %	no LAA thrombus (*n* = 181) %	*p*-value
Chicken-wing	59	7 (12)	52 (88)	*p* < 0.05
Non-chicken-wing	172	43 (25)	129 (75)	0.658
windsock	119	31 (26)	88 (74)	0.111
cactus	36	8 (22)	28 (78)	1.000
cauliflower	17	4 (24)	13 (76)	0.767

### Neuro-embolic events

Within the seven patients with chicken-wing configuration and LAA thrombus, 42.9% (*n* = 3) experienced a neuro-embolic event within 18 months after TAVI. In contrast, 20.9% (*n* = 9) of the 43 patients with non-chicken-wing configuration and LAA thrombus, experienced a neuro-embolic event within 18 months after TAVI (OR: 0.35, 95%CI 0.67 to 1.87, *p* = 0.34).

## Discussion

This is the first study to analyse different LAA morphologies and the correlation to LAA thrombus and neurological thromboembolic events in patients with aortic stenosis and atrial fibrillation. Our data show that LAA morphology may impact the development of a thrombus to an embolus which may influence neurological outcome. In our study, chicken-wing morphology is associated with a significantly lower thrombus rate. Nevertheless, the risk of a neuro-embolic event in the presence of a thrombus was doubled in patients with chicken-wing morphology.

The relationship between LAA morphology and stroke is of great relevance, especially in patients with atrial fibrillation. Previous data are ambivalent [3, 7–16]. Several studies analysed a correlation between LAA morphology and an increased risk of stroke [7–9, 11–18]. While Di Biase et al. [9] observed that non-chicken-wing was the most prevalent morphology type in stroke patients, Kosiuk et al. [10] showed that chicken-wing morphology is associated with higher periprocedural thromboembolic risk in patients undergoing AF ablation.

The present study observed that LAA morphology impacts both thrombus prevalence and the risk of neuro-embolic events. These results are novel and could be clinically relevant, especially for the discussion of further anticoagulation. Potentially, thrombus patients with chicken-wing morphologies would benefit from a more aggressive antithrombotic therapy compared to those with non-chicken-wing morphology.

Conflicting results may be explained by different LAA classifications with overlapping morphologies [7, 9, 15]. Previous studies did not discriminate between the risk of thrombus formation and resulting neuro-embolic events [7–9, 11–14, 16–18]. This could explain their inconsistent results for the association between LAA morphology and stroke.

In addition, most studies examine LAA thrombus in retrospective populations after strokes or TIA [8, 9, 13, 14], which may overestimate the stroke risk and underestimate the rate of asymptomatic LAA thrombi.

Furthermore, several studies used *non*-ECG-gated CT scans, which are ineligible for correct identification of the LAA morphology and the diagnosis of a LAA thrombus [13, 16].

In addition, our data confirm the results of Palmer et al. [19] for a larger collective and with an exclusive focus on patients with atrial fibrillation. Correspondent to their findings, we observed a high percentage of LAA thrombus in patients before TAVI. However, this study included patients with and without atrial fibrillation, which may bias the thrombus rates. Furthermore, our follow-up contained 18 months, whereas the follow-up of Palmer et al. [19] contained only the time of index admission and the authors do not present their median follow-up period.

### Anatomic approach

The LAA derives from the primordial left atrium with a reservoir function in conditions of fluid overload [20]. In patients with non-valvular atrial fibrillation, it is with over 90% the major location for intra-cardiac thrombus [2, 20]. Previous studies suggest that the LAA morphology correlates with the risk of thrombus and stroke [3, 7–9, 13, 16]. However, no statistical discrimination between both risks is available so far. The present study suggests that the LAA morphology might have an impact on the development from a LAA thrombus into an embolus with resulting neuro-embolic event. In our study, patients with chicken-wing morphology had a lower risk to develop a LAA thrombus. However, once a thrombus is formed patients with chicken-wing LAA are more likely to develop a neuro-embolic event.

Published LAA-classification schemes show inter-observer and intra-observer variabilities with limited reproducibility [7, 9, 15]. Our data suggest that it is crucial to differentiate chicken-wing and windsock morphologies. Since windsock showed the highest thrombus-rates and chicken-wing, the highest neuro-embolic event rate in patients with LAA thrombus. Whereas, from our experience, both chicken-wing and windsock configuration are easiest to detect. Correct identification of these two configurations could be a useful tool for radiologists.

### Study limitations

The main limitation was the monocentric study design. Moreover, our highly specific patients population with AS and atrial fibrillation, results in relatively small number of patients. In addition, common LAA-classification schemes show inter-observer and intra-observer variabilities with limited reproducibility.

## Conclusion

Our study suggests that patients with chicken-wing morphology, together with atrial fibrillation and AS, have a significantly lower risk of LAA-thrombus formation. However, in patients with both chicken-wing configuration and thrombus, the risk of neuro-embolic events is significantly higher compared to non-chicken-wing morphologies. This underlines the importance of LAA evaluation in thoracic CT scans. These results could also have an impact on anticoagulation management in patients with atrial fibrillation. However, further prospective studies are needed to confirm these conclusions.

## Data Availability

The data presented in this study are available on request from the corresponding author.
